# tRNA-Derived Small Non-Coding RNAs in Response to Ischemia Inhibit Angiogenesis

**DOI:** 10.1038/srep20850

**Published:** 2016-02-11

**Authors:** Qing Li, Bin Hu, Guo-wen Hu, Chun-yuan Chen, Xin Niu, Juan Liu, Shu-min Zhou, Chang-qing Zhang, Yang Wang, Zhi-Feng Deng

**Affiliations:** 1 Institute of Microsurgery on Extremities, Shanghai Jiaotong University Affiliated Sixth People’s Hospital, Shanghai 200233, China; 2 Department of Neurosurgery, Shanghai Jiaotong University Affiliated Sixth People’s Hospital, Shanghai 200233, China; 3 Department of Orthopedic Surgery, Shanghai Jiaotong University Affiliated Sixth People’s Hospital, Shanghai 200233, China; 4 Graduate School of Nanchang University, Nanchang 330006, China

## Abstract

Ischemic injuries will lead to necrotic tissue damage, and post-ischemia angiogenesis plays critical roles in blood flow restoration and tissue recovery. Recently, several types of small RNAs have been reported to be involved in this process. In this study, we first generated a rat brain ischemic model to investigate the involvement of new types of small RNAs in ischemia. We utilized deep sequencing and bioinformatics analyses to demonstrate that the level of small RNA fragments derived from tRNAs strikingly increased in the ischemic rat brain. Among these sequences, tRNA^Val^- and tRNA^Gly^-derived small RNAs account for the most abundant segments. The up-regulation of tRNA^Val^- and tRNA^Gly^-derived fragments was verified through northern blot and quantitative PCR analyses. The levels of these two fragments also increased in a mouse hindlimb ischemia model and cellular hypoxia model. Importantly, up-regulation of the tRNA^Val^- and tRNA^Gly^-derived fragments in endothelial cells inhibited cell proliferation, migration and tube formation. Furthermore, we showed that these small RNAs are generated by angiogenin cleavage. Our results indicate that tRNA-derived fragments are involved in tissue ischemia, and we demonstrate for the first time that tRNA^Val^- and tRNA^Gly^-derived fragments inhibit angiogenesis by modulating the function of endothelial cells.

Ischemic injuries are due to the interruption of or an insufficient blood supply and can lead to a loss of function in ischemic organs. For example, ischemic stroke is a leading cause of disability and death among the adult population in response to cerebrovascular insults. After ischemic stroke, ischemia and anoxia result in the degeneration and necrosis of neurons^1^. Numerous studies showed that angiogenesis accompanies ischemic stroke, wherein the expression of angiogenic factors is induced within hours, and the microvascular density increases within days of the onset of ischemia^2^,^3^. Post-ischemia angiogenesis serves as a key step for neurovascular repair and stroke recovery by improving cerebral perfusion, promoting the generation of new neurons and their migration to the ischemic zone, and facilitating neuronal plasticity and remodeling^4^,^5^. Because revascularization plays a crucial role in promoting the recovery of ischemic tissues, the molecular mechanisms underlying this process need to be fully elucidated.

Small non-coding RNAs are defined as transcripts of <200 nucleotides (nts) that lack protein-coding capacity^6^; this class of RNAs includes microRNAs (miRNAs), piwi-interacting RNAs (piRNAs), small nucleolar RNAs (snoRNAs), and transfer RNAs (tRNAs)^7^. Among others, miRNAs have been established to be critical for vascular development, physiology and disease. Previous studies^8^,^9^,^10^,^11^,^12^ showed that various miRNAs are highly expressed in vascular endothelial cells (ECs) and/or in tumor cells. These miRNAs serve as critical regulators of angiogenesis by modulating the expression of several angiogenic or anti-angiogenic factors and affecting the endothelial differentiation of pluripotent stem cells, ECs proliferation and migration^8^,^9^,^10^,^11^,^12^. PiRNAs are another class of small non-coding RNAs that are involved in this process. A recent study published by Yan H and colleagues^13^ demonstrated that piRNA-823 can act a positive regulator of neovascularization to promote tumorigenesis. This initial evidence for a functional role of piRNAs in angiogenesis implicates the importance of another class of small non-coding RNAs in this process.

tRNAs are adaptor molecules that are 73 to 94 nts in length and act as an indispensable component in the synthesis of new proteins^14^. In addition to their functional role in protein translation, tRNAs perform non-canonical functions in cellular communication, reverse transcription, the targeting of proteins for degradation, etc^14^,^15^,^16^,^17^,^18^,^19^. Recently, a new class of small RNAs distinct from canonical small RNAs has been discovered in various organisms^20^,^21^. In 2005, Fu *et al.*^22^ unexpectedly discovered a significant number of sequences that were cleaved from the 5′ end of tRNAs at the anti-codon loop. Therefore, these tRNA-derived fragments were named tRNA halves^20^,^22^. Subsequent studies showed that various stress conditions can induce tRNA cleavage, and these fragments can serve as small interfering RNAs that regulate the activity of translation factors^22^,^23^,^24^. They function as modulators in diverse biological processes, including cell proliferation and apoptosis^19^,^20^,^25^. However, few studies have investigated the involvement of tRNA-derived small RNAs in ischemic pathophysiology and their roles in post-ischemia angiogenesis.

In the present study, we profiled small RNA transcripts of <35 nts from rat ischemic right brain and uninjured left brain tissues using Solexa deep sequencing. Surprisingly, we discovered that a class of small RNAs that primarily were 29 to 34 nts in length were significantly up-regulated after ischemia. Using a bioinformatics analysis, we found that an overwhelming majority of these small RNAs were derived from tRNA. Most of the sequences were selectively cleaved from the 5′ end of their parental mature tRNA. We then focused on the two most abundant small RNAs derived from tRNA^Val^ and tRNA^Gly^ for further analysis. We utilized Northern blotting and quantitative RT-PCR analyses to confirm that these two small RNA families were not only increased in ischemic brain tissues but also remarkably induced in a hindlimb ischemia and cell hypoxia model. Importantly, we showed that these tRNA-derived fragments could inhibit the proliferation, migration, and tube formation of ECs. Our results suggest that these tRNA-derived small RNAs function as negative regulators of angiogenesis.

## Results

### tRNA-derived small RNAs were extremely enriched in the post-ischemic rat brain

**Figure 1:**
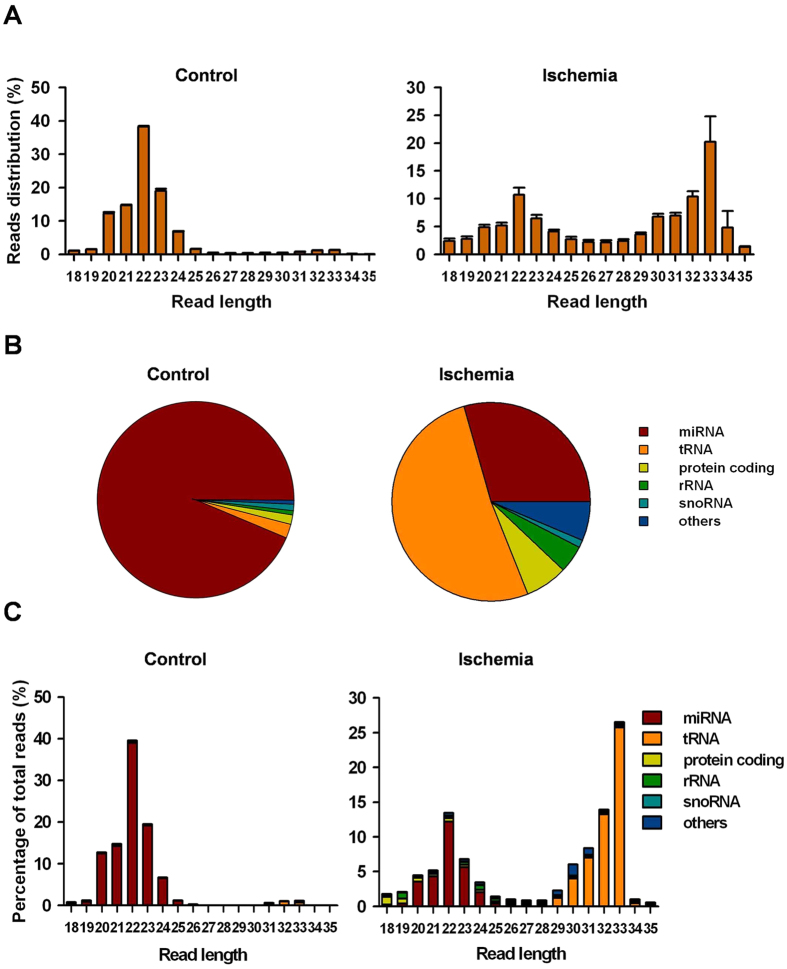
tRNA-derived fragments were highly enriched in ischemic rat brain. Adult male Sprague-Dawley rats were used to establish the model of middle cerebral artery occlusion (MCAO). Twenty-four hours later, the uninjured left brains and ischemic right brains from 5 rats were selected for deep sequencing. (**A**) Length distributions of small RNAs in the uninjured left brain (Control) and ischemic right brain (Ischemia). (**B**) Catalogue of small RNA populations in control and ischemic brain. (**C**) TRNA-derived fragments comprised the majority of 29–34 nts small RNA population in the ischemic right brain.

To investigate the involvement of small RNAs in tissue ischemia, we first generated the rat MCAO model. Twenty-four hours later, the ischemic right brain tissues were processed for small RNA (<35 nts) deep sequencing, and the uninjured left brain tissues served as controls. In the control group, most small RNAs distributed at a read length of 20–24 nts and peaked at 22 nts. Although many small RNAs in the ischemic group were also 20–24 nts in length, most reads indicated a length of 29–34 nts (Fig. 1A). Further analyses showed that the majority of small RNAs (93.7%) in the control group were annotated as miRNAs (Fig. 1B) and mainly distributed at 20–24 nts (Fig. 1C). The remaining RNAs were fragments from tRNA, protein coding RNAs, rRNAs, snoRNAs and other types of small RNAs. In the ischemic group, the most abundant small RNAs were fragments derived from tRNAs (51.6%) (Fig. 1B), which accounted for the majority of the 29–34 nts small RNA population (Fig. 1C). These results indicated that tRNAs are cleaved into small fragments during ischemia, suggesting that these tRNA-derived fragments may play important roles in brain tissue ischemia.

### The characterization of up-regulated tRNA-derived small RNAs

**Figure 2:**
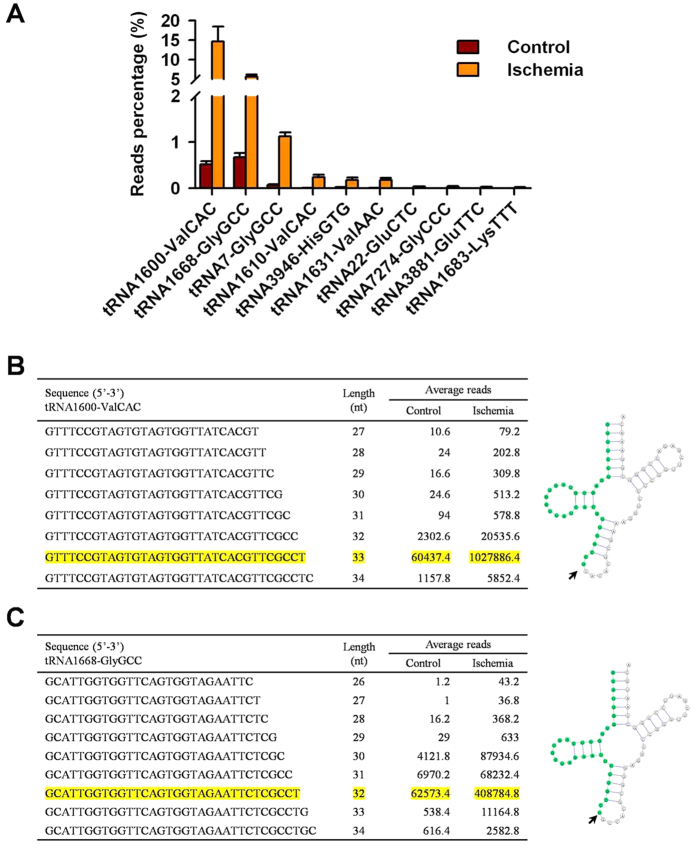
Sequence analysis of tRNA-derived fragments. The sequences of tRNA-derived fragments were matched to the Genomic tRNA Database, and the top 10 tRNAs with highest read numbers are shown in (**A**). Alignments of tRNA^Val^ (**B**) and tRNA^Gly^ (**C**) derived fragments; the most popular sequence for each tRNA is highlighted. The arrow head indicates the cleavage site.

**Figure 3:**
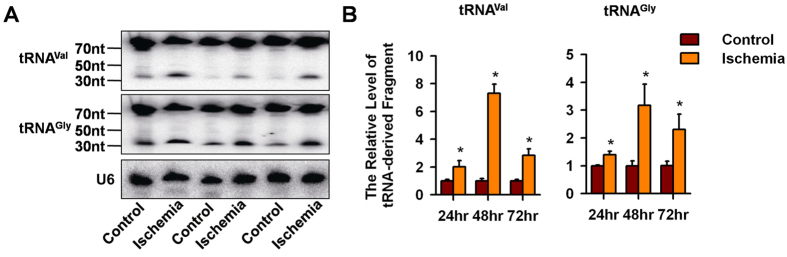
Verification of tRNA-derived fragments in rat brain. (**A**) Northern blot analysis showed that the level of tRNA^Val^- and tRNA^Gly^-derived fragments increased 24 hrs after MCAO. U6 were used as a loading control. (**B**) qPCR analysis of the level of tRNA^Val^- and tRNA^Gly^-derived fragments in the rat brain 24 hr, 48 hr, and 72 hr after ischemia induction. *P < 0.05.

Next, we characterized these up-regulated small RNA fragments by comparing them to the Genomic tRNA Database. The results showed that most of these fragments in the ischemic group were derived from tRNA^Val(CAC)^ and tRNA^Gly(GCC)^, and the top 10 matched tRNAs are shown in Fig. 2A. Low levels of tRNA-derived fragments were also observed in the control group (Fig. 2A, supplementary Table 1). A further sequence analysis showed that most of the fragments that matched the 5′ half of the specific tRNA shared identical 5′ sequences and only differed by a few nucleotides at the 3′ ends; furthermore, they were preferentially cleaved at the anti-codon loop (Fig. 2B,C, supplementary Table 2). These data suggest that these tRNA-derived small RNAs are not random products of tRNA degradation but the result of accurate cleavage modulations in response to hypoxia. We then used Northern blot to verify the up-regulation of tRNA^Val(CAC)^- and tRNA^Gly(GCC)^-derived fragments. The results showed that two bands were detected for each tRNA (73 nts and 33 nts for tRNA^Val(CAC)^, 71 nts and 32 nts for tRNA^Gly(GCC)^, and the level of the smaller fragments in the ischemia group increased compared with that of the control group (Fig. 3A). Moreover, qPCR analyses showed that the levels of these tRNA-derived fragments were significantly elevated in the rat brain 24 hr (1.5 and 1.4 folds), 48 hr (7.3 and 3.2 folds), and 72 hr (2.8 and 2.3 folds for tRNA^Val(CAC)^- and tRNA^Gly(GCC)^-derived fragments respectively) after ischemia induction compared with that of the uninjured brain tissue (Fig. 3B).

### tRNA cleavage widely occurred during ischemic-hypoxic conditions

**Figure 4:**
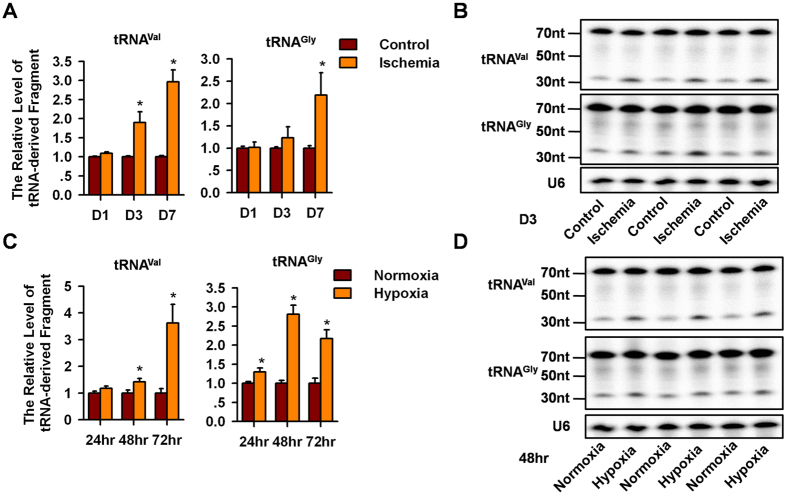
Verification of tRNA-derived fragments in the mouse hindlimb ischemia model and endothelial cell hypoxia model. (**A**) qPCR analysis of tRNA^Val^- and tRNA^Gly^-derived fragments in samples from mouse hindlimb at the indicated time after ischemia. D1, day1; D3, day3; D7, day7. *P < 0.05. (**B**) Northern blot analyses of tRNA^Val^- and tRNA^Gly^-derived fragments in the mouse hindlimb 3 days after ischemia. (**C**) The cell hypoxia model was generated by culturing ECs in 1% oxygen, and the RNA was harvested at the indicated time. qPCR analysis of the level of tRNA^Val^- and tRNA^Gly^-derived fragments. *P < 0.05. (**D**) Northern blot analysis of tRNA^Val^- and tRNA^Gly^-derived fragments in ECs 48 hrs after hypoxia.

The mouse hindlimb ischemia models were generated to determine whether tRNA cleavage is widespread in ischemic-hypoxic conditions, and the tissues were harvested at D1, D3 and D7. The levels of tRNA^Val(CAC)^- and tRNA^Gly(GCC)^-derived fragments in the control and ischemic hindlimb tissues were assessed using qPCR analysis. After the induction of ischemia, the level of tRNA^Val(CAC)^-derived small RNAs at D3 and D7 (1.9 and 3 folds respectively) and the level of tRNA^Gly(GCC)^-derived fragments at D7 (2.2 folds) (Fig. 4A) significantly increased in ischemic tissues compared to the levels in the control group. The levels of small RNAs derived from tRNA^Gly(GCC)^ also increased at D3 (1.2 folds) although with no statistical difference. Northern blot further confirmed the increase of these fragments at D3 (Fig. 4B). The result indicates that these tRNA-derived small RNAs are responsive to ischemic stimuli and may have regulatory roles in ischemic pathophysiology.

Ischemia is a restriction of the blood supply, which starves tissues of oxygen and nutrition. Post-ischemia angiogenesis has been accepted as a key step in the recovery from ischemic disorders^4^,^5^. ECs hypoxia models were established to assess the level of tRNA-derived small RNAs to investigate the involvement of tRNA-derived fragments in angiogenesis. ECs were cultured under normoxic or hypoxic (1.0% O_2_) conditions for 24 hr, 48 hr, and 72 hr, respectively. The levels of tRNA^Val(CAC)^- and tRNA^Gly(GCC)^-derived small RNAs were determined using qPCR analyses. The results revealed that the level these tRNA-derived small RNAs dramatically increased in ECs after hypoxic induction for 48 hr (1.4 and 2.8) and 72 hr (3.6 and 2.2 folds for tRNA^Val(CAC)^- and tRNA^Gly(GCC)^-derived fragments respectively) (Fig. 4C). The up-regulation of these small RNAs at 48 hr post hypoxia were further confirmed by northern blot analysis (Fig. 4D). This finding suggests that these hypoxia-responsive small RNAs may act as important regulators of angiogenesis by modulating the function of ECs.

### tRNA-derived small RNAs could function as negative regulators in angiogenesis

**Figure 5:**
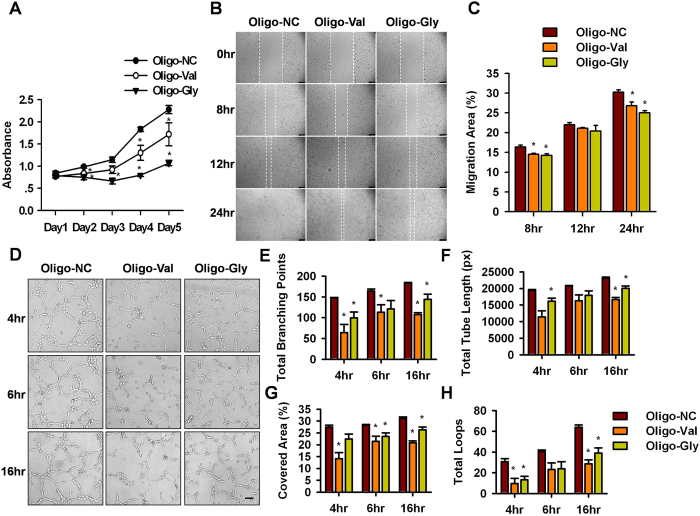
Functional analyses of tRNA-derived fragments in endothelial cells. HUVEC were transfected with Oligo-NC, Oligo-Val, and Oligo-Gly for 24 hrs and then subjected to the following analyses. (**A**) Transfection of Oligo-Val and Oligo-Gly in HUVEC inhibited cell proliferation as analyzed by CCK8. *P < 0.05. (**B**) The up-regulation of tRNA^Val^- and tRNA^Gly^-derived fragments hindered the migration of HUVEC. Scale bar: 250 μm. (**C**) Statistical analysis of cell migration. The migration capacity was assessed by measuring the scratch closure area at each time point. *P < 0.05. (**D**) The *in vitro* angiogenesis assay shows that the up-regulation of tRNA^Val^- and tRNA^Gly^-derived fragments suppressed the tube formation of HUVEC. Scale bar: 250 μm. (**E–H**) The branching points, tube loops, tube covered area and tube length per field were counted to quantify the tube formation ability of the cells. *P < 0.05

To explore the functional roles of tRNA^Val(CAC)^- and tRNA^Gly(GCC)^-derived fragments in angiogenesis, the small fragments were synthesized and individually transfected into ECs (denoted as Oligo-Val and Oligo-Gly). Twenty-four hours later, the level of tRNA^Val(CAC)^- and tRNA^Gly(GCC)^-derived small RNAs was determined by qPCR analysis (Fig. S1). The results showed that there was a 4- and 66- folds increase for tRNA^Val(CAC)^- and tRNA^Gly(GCC)^-derived small RNAs, respectively. Then the cell proliferation, migration, and tube formation capacities were assessed using a series of *in vitro* angiogenesis assays. Strikingly, the CCK-8 cell counting analysis showed that the up-regulation of Oligo-Val and Oligo-Gly in ECs significantly inhibited cell proliferation (Fig. 5A). A scratch wound assay was then utilized to determine the effect of tRNA^Val(CAC)^- and tRNA^Gly(GCC)^-derived fragments on the migration of ECs. Transfection with Oligo-Val or Oligo-Gly slightly reduced the migration compared with those transfected with Oligo-NC, as determined by the migration area (Fig. 5B,C). To determine their effects on tube formation, the cells were seeded on Matrigel and cultured for 4 hr, 6 hr, or 16 hr. The total branching points, total tube length, tube covered area, and total loops at the indicated time were measured to quantify the ability of ECs to form tubes. Compared with the control group, all indicators that evaluated the capability of ECs to form tubes were attenuated in ECs transfected with Oligo-Val (Fig. 5D–H), indicating that the up-regulation of tRNA^Val(CAC)^- and tRNA^Gly(GCC)^-derived fragments in ECs could inhibit tube formation. These data suggest that the tRNA^Val(CAC)^- and tRNA^Gly(GCC)^-derived small RNAs play negative roles in angiogenesis by inhibiting the proliferation, migration, and tube formation of ECs.

### Angiogenin is responsible for the cleavage of tRNA upon hypoxia

**Figure 6:**
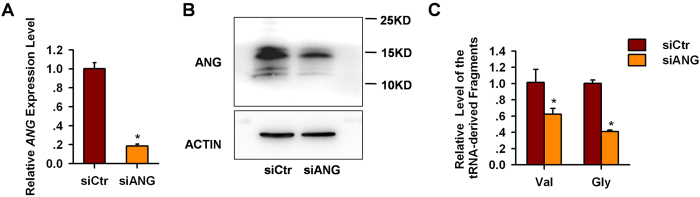
Angiogenin is required for the cleavage of tRNA upon hypoxia. ECs were transfected with 50 nM siCtr or siANG. Twenty-four hours later, the cells were replated, and a second siRNA transfection was performed. Then the cells were subjected to hypoxia (1% oxygen) for 48 hrs. RNA and protein were harvest for analyses. (**A**) qPCR analysis showed that the mRNA level of ANG decreased in siANG transfection group. siCtr, RNAi control. *P < 0.05. (**B**) Transfection of siANG reduced the protein level of ANG. (**C**) qPCR analysis showed that the level of tRNA^Val^- and tRNA^Gly^-derived fragments decreased in the cells transfected with siANG compared with that transfected with siCtr. *P < 0.05.

Previous studies demonstrated that tRNAs are natural substrates of angiogenin (ANG)^26^,^27^, which is a member of the RNase A superfamily and can cleave tRNA at the anti-codon region to produce tRNA halves^27^,^28^,^29^,^30^ in the cytoplasm. To investigate whether the tRNA-derived small RNAs in response to hypoxia were also generated by ANG, we first analyzed the localization of ANG. The result showed that ANG localized to granules in the cytoplasm upon hypoxia stimulation (Fig. S2). Then we used siRNA to knock down ANG expression in endothelial cells during hypoxia. Transfection of siANG efficiently reduced the mRNA expression of ANG by 82%, and protein level of ANG also significantly decreased (Fig. 6A,B). By qPCR analysis, we showed that the production of tRNA^Val(CAC)^- and tRNA^Gly(GCC)^-derived fragments were reduced by 38% and 59% respectively upon the down-regulation of ANG (Fig. 6C). This finding indicates that ANG specifically cleave these tRNA in response to hypoxia.

## Discussion

Ischemic stroke is a common clinical disorder and generally causes vascular and neuronal damage, both of which affect the extent of ischemic injury and post-stroke outcome^7^. After the onset of ischemia, the ischemic brain undergoes a continuum of molecular responses that determine the regeneration and function of ischemic tissue. Previous studies demonstrated that the expression profiles of different classes of small non-coding RNAs, including miRNAs and piRNAs, were changed in cerebral ischemia^31^,^32^,^33^. Our present study showed that focal ischemia altered the expression profile of small RNAs transcripts of <35 nts in the rat brain. A class of tRNA-derived small RNAs (29 to 34 nts) were extremely enriched in post-ischemic tissues, among which small RNAs cleaved from tRNA^Val(CAC)^ and tRNA^Gly(GCC)^ were the most abundant. We further showed that these two small RNA families were widely up-regulated in ischemic injuries, suggesting that they may have important roles in ischemic pathophysiology. Most importantly, we found that the up-regulation of these two small RNA families could profoundly inhibit the proliferation, migration, and tube formation of ECs. To the best of our knowledge, this study is the first to show that ischemic-hypoxic injuries induce the cleavage of specific tRNAs and that these tRNA-derived small RNAs can act as signaling molecules to regulate angiogenesis by modulating the function of ECs.

Small RNAs derived from tRNA specific cleavage have recently gained significant attention. Currently, the small RNAs derived from tRNAs can be categorized as follows: those from the 5′ end of tRNAs, those from the 3′ end of mature tRNAs, and those from the 3′ end of pre-tRNAs^34^. In the present study, we found that a class of tRNA-derived small RNAs accumulated in post-ischemic tissues. The tRNA cleavage products originated from the 5′ end of the corresponding tRNAs, with cleavage sites located preferentially at the anti-codon loop (32 to 33 nts from the 5′ end). We obtained few fragments derived from the 3′ end sequence of tRNA. Because a number of recent reports detailed the sequencing and analysis of 3′ tRNA-derived fragments of approximately 20 nts in size^20^,^35^,^36^,^37^,^38^, we hypothesized that some molecules may easily and rapidly degrade the small RNAs derived from the 3′ end of tRNA. The ultra-high enrichment features, along with their preferential cleavage and length distributions, strongly support that these sequences are not random products of tRNA degradation, but rather are the result of strictly regulated cleavage. Our results showed that knockdown the expression of ANG during hypoxia resulted in the reduction of the tRNA-derived fragments. Together with previous studies^26^,^27^, these data indicates that tRNAs would be cleaved specifically by ANG under stress condition.

Previously, several studies showed that stress conditions can significantly up-regulate the production of small RNAs derived from the 5′ end of tRNA^23^,^26^,^39^,^40^. Fu and his colleagues^26^ reported that heat shock, hypothermia, hypoxia and irradiation can induce the production of fragments derived from tRNAs in nutritionally deficient human tumor cells, including tRNA^Gly(GCC)^, tRNA^Val(AAC)^, tRNA^Met(CAT)^, etc. Gong *et al.*^39^ showed that rickettsia infection significantly up-regulated the levels of tRNA^Gly(GCC)^- and tRNA^Val(GTG)^-derived small RNAs in both mouse tissues and human cells. In liver biopsies from subjects with chronic hepatitis and hepatocellular carcinoma, the level of small RNAs derived from tRNA^Gly^ and tRNA^Val^ also increased^40^. In this study, a variety of 5′ tRNA-derived small RNAs were profiled in response to ischemia, and the top four small RNA families were each cleaved from tRNA^Val(CAC)^, tRNA^Gly(GCC)^, tRNA^His(GTG)^, and tRNA^Val(AAC)^. tRNA^Gly(GCC)^ and tRNA^Val(AAC)^ are widely cleaved in response to stress. Moreover, the small RNAs from tRNA^Val(CAC)^ were the most abundant in our research, suggesting that the tRNA cleavage may depend on the conditions.

Over the past decade, intriguing studies showed that rather than some nonfunctional degradation products of tRNA, these tRNA-derived small RNAs are produced in a controlled fashion and serve as novel signaling molecules in the response to stress^20^,^41^,^42^,^43^. The small RNAs derived from tRNA^Ala^ and tRNA^Cyc^ in stressed cells can cause translational repression and activate a cytoprotective stress response program by stimulating the formation of stress granules (SGs) in the stress response of cells^23^,^24^, which may promote cell survival under stress conditions^42^. Severe or chronic stress induces apoptosis, which involves cytochrome *c* (Cyt *c*) release from mitochondria and subsequent apoptosome formation^43^. Saikia *et al.*^43^ showed that the small RNAs derived from tRNA can interact with Cyt *c* to protect cells from apoptosis during osmotic stress. In addition, tRF-1001, which is derived from the 3′ end of a tRNA^Ser(TGA)^ precursor, can positively regulate cell proliferation to promote tumor growth^20^. A variety of physiological and pathological situations, including hypoxia, ischemia, and tumor development, are thought to be able to rapidly initiate angiogenesis. In this study, we investigated the function of tRNA-derived small RNAs in angiogenesis for the first time. Our results showed that the tRNA^Val(CAC)^- and tRNA^Gly(GCC)^-derived small RNAs suppress angiogenesis by inhibiting the proliferation, migration and tube formation of ECs. Since post-ischemia angiogenesis plays crucial role for tissue repair and function recovery, the increase of these fragments in ischemic tissues may impede angiogenesis and prevent or slow down tissue regeneration. Therefore, we speculated that eliminating these small fragments may promote angiogenesis in ischemic tissue and facilitate organ function recovery. Together with previous reports, our data indicate that tRNA-derived fragments are involved in diverse biological processes. However, determining the exact molecular mechanism warrants further investigation.

## Methods

The methods were carried out in accordance with the approved guidelines.

### Cell culture and RNA oligo transfection

Human umbilical vein endothelial cell (HUVEC) (Life Technologies Corporation) were maintained in Medium200 supplemented with Low Serum Growth Supplement (LSGS) and 10% FBS. The cells were cultured in a hypoxic gas mixture (1% O_2_, 5% CO_2_, and balance N_2_) to induce hypoxia, whereas the normoxic cells were placed at 37 °C in a 21% O_2_, 5% CO_2_, and 74% N_2_ humidified incubator until harvest. HUVEC cells were seeded at a density of 2 × 10^5^ cells/ml and cultured for 24 h, after which Oligo-NC (5′-UUCUCCGAACGUGUCACGUTT-3′), Oligo-Val (5′-GUUUCCGUAGUGUAGUGGUUAUCACGUUCGCCU3′) and Oligo-Gly (5′-GCAUUGGUGGUUCAGUGGUAGAAUUCUCGCCU-3′) were transfected at the final concentration of 50 nM using oligofectamine reagent (Invitrogen) according to the manufacturer’s protocol.

For siRNA transfection, HUVECs were transfected with 50 nM of siRNA. On the next day, the cells were replated, and a second siRNA transfection was performed, after which cells were cultured in a hypoxic gas mixture for 48 hrs. The ANG siRNA (siGENOME SMART pool, M-011206-01-0005) were purchased from GE Healthcare Dharmacon Inc.

### Generation of rat focal cerebral ischemia model

All care and handling of animals was performed with the approval of the Ethical Review Board of Shanghai Six people’s Hospital affiliated of Shanghai Jiaotong University. The animals were raised in a specific pathogen-free (SPF) environment. Adult male Sprague-Dawley rats (250 to 300 g) were used to establish the middle cerebral artery occlusion (MCAO) model as previously described^44^. After anesthetization, the carotid arteries were exposed, and a 4-0 nylon intraluminal suture was introduced from the minimal incision of the external carotid artery into the internal carotid artery to block the origin of the MCA. After 2 hours of occlusion, reperfusion was performed by withdrawing the suture. Twenty four hours later, the uninjured left brains and ischemic right brains from 5 rats displaying a significant neurologic deficit of the left forelimb according to the Longa 5-point scale^45^ were selected for deep sequencing.

### Generation of mouse hind limb ischemia model

The murine model of hind limb ischemia was generated by ligating the common left iliac-femoral artery. The contralateral limb served as an internal control. After the induction of hindlimb ischemia, the morphology of the limb was determined and the blood flow of the ischemic and normal limb was measured using a laser Doppler blood flow meter. The mice were then sacrificed at indicated time.

### RNA extraction, small RNA library preparation and sequencing

The total RNA was isolated from mouse tissue and cultured cells using the TRIzol Reagent (Invitrogen) according to the instructions of the manufacturer. The total RNA of the rat brain for sequencing was prepared using the mirVana™ miRNA Isolation Kit (Ambion). The Shanghai BioChip Company constructed the small RNA library and carried out the Solexa high-throughput sequencing following their standard protocols. Briefly, 5′- and 3′- adapters were ligated to the obtained small RNA. Reverse transcription followed by PCR was used to create cDNA constructs. Subsequently, a 145 nts to 162 nts fraction corresponding to approximately the adapter-ligated constructs derived from the 20 nts to 35 nts small RNA fragments was excised and purified. The purified libraries were quantified with the Qubit® 2.0 Fluorometer and validated using the Agilent 2100 bioanalyzer to confirm the insert size and calculate the molar concentration. The library that passed quality control was sequenced on an Illumina Hi-Seq 2000 apparatus. Small RNA-sequencing data was deposited on GEO (GSE70473).

### Data processing and analysis

The raw Solexa sequencing data were refined using fastx (fastx_toolkit-0.0.13.2), which included filtering out low quality reads and short reads (<18 nts). The small RNA clean reads were first compared to the Sanger miRBase database (http://www.mirbase.org/) to identify known miRNAs, and the unidentified sequences were further aligned to several other small RNA databases, including the ncRNA Database, piRNA Database and Rfam Database, while allowing for two mismatches. The sequences of tRNA-derived fragments were matched to the Genomic tRNA Database (http://gtrnadb.ucsc.edu/) to obtain their position information and potential cleavage sites.

### Northern blot

The total RNAs extracted from the rat brain were resolved on a 15%, 7M urea polyacrylamide gel. The RNA was transferred from the gel to the NC membrane (PerkinElmer) at 250 mA for approximately 3 hours using a semi-dry transfer apparatus (BioRad Transblot). The NC membrane was air-dried at RT for 10 min. The membrane was cross-linked at 1200 mJ using a UV crosslinker. The membrane was pre-hybridized in PerfectHyb plus solution (Sigma-Aldrich) at 45 °C overnight. The probes were labeled with [γ-^32^P]-ATP (PerkinElmer) using T4 PNK (Fermentas) and added to the pre-hybridization buffer for hybridization at 48 °C for 6 hours. The membrane was washed 3–5 times with low-stringent washing buffer, 10X SSC, and then several times with high-stringent washing buffer (2X SSC, 1 time; 1X SSC, 1 time; 0.5X SSC,1 time). The radioactive signal was then obtained via autoradiography. The membrane was stripped in 1% SDS at 85 °C for 1 hour before being hybridized again. The tRNA^Val^ probe sequence was 5′-AGGCGAACGTGATAACCACTACACTACGGAAAC-3′; the tRNA^Gly^ probe sequence was 5′-AGGCGAGAATTCTACCACTGAACCACCAATGC-3′; the U6 snRNA probe sequence was 5′-GAATTTGCGTGTCATCCTTGCGCAGGGGCCATGCTAA -3′.

### Small RNA real time quantitative PCR (qPCR).

Small RNAs were reverse transcripted with specific stem-loop primers for tRNA^Val^- or tRNA^Gly^-derived fragments using miRcute miRNA First-Strand cDNA Synthesis Kit (Tiangen) following the manufacturer’s instructions. The RT primer for tRNA^Val^-derived fragments was 5′- GACCAGCGACCGTGTCGTGGAGTCGGCTAATGGTCGCTGGTCAGGCGAAC -3′; the RT primer for tRNA^Gly^-derived fragments was 5′- GACCAGCGACCGTGTCGTGGAGTCGGCTAATGGTCGCTGGTCAGGCGAG -3′. Realtime qPCR amplification was performed using FS Universal SYBR Green Master (Rox) on the ABI 7900 realtime PCR detection system. U6 was used for normalization. qPCR reactions for all samples were performed in triplicate. The forward Primer sequence for tRNA^Val^-derived fragments was 5′- CTCGCAGGCGTTTCCGTAGTGTAGTGGT -3′; the forward Primer sequence for tRNA^Gly^-derived fragments was 5′- CTCCGTGACCGCATTGGTGGTTCAG -3′.

### Cell proliferation assay

A Cell Counting Kit-8 assay (CCK-8; Dojindo) was used to assess cell proliferation. Briefly, HUVECs cells were transfected with oligo and then seeded at 5 × 10^4^ cells/ml in a 96-well plate. Subsequently, 10 μL of CCK-8 solution was added to the HUVECs, and the cells were incubated for 3 hours at 37 °C. The absorbance was measured at 450 nm using a microplate reader. The cells were analyzed for 5 days.

### Cell migration assay

The scratch wound assay was used to analyze the effect of Oligo-Val and Oligo-Glyon the migration of endothelial cells. Briefly, after transfection with Oligo-Val and Oligo-Gly, 2 × 10^5^ HUVEC cells were seeded in 12-well plates and maintained at 37 °C to permit cell adhesion and the formation of a confluent monolayer. These confluent monolayers were then scratched using a 200 μL pipette tip. The medium was removed and rinsed once with PBS to remove the debris and smooth the edge of the scratch, and the PBS was then replaced with fresh medium. Wound closure was detected at indicated time. The wound closure was analyzed using the MetaMorph software (Molecular Devices), and the wound area at each time point was normalized to its corresponding area at 0 hr.

### Tube formation assay

Twenty-four hours after transfection, HUVECs (7 × 10^4^) were cultured in a 12-well plate coated with Matrigel Basement Membrane Matrix (BD Biosciences). Tube formation was quantified at 4 hr, 6 hr, and 18 hr. The total branching points, total tube length, covered area, and total loops per image were measured by a blinded independent observer.

### Antibodies

Antibodies against ANG or ACTIN were obtained from Thermo Fisher Scientific, Inc. and Abcam, plc., respectively.

### Statistical analysis

All of the experiments were performed at least three times. The reported data are means ± SEM. An unpaired Student’s t-test was used to statistically compare the data. P values <0.05 were considered statistically significant.

## Additional Information

**How to cite this article**: Li, Q. *et al.* tRNA-Derived Small Non-Coding RNAs in Response to Ischemia Inhibit Angiogenesis. *Sci. Rep.* **6**, 20850; doi: 10.1038/srep20850 (2016).

## Supplementary Material

Supplementary Information
