# Supplementary material for: tRNA-Derived Small Non-Coding RNAs in Response to Ischemia Inhibit Angiogenesis
Source: Sci Rep. 2016 Feb 11;6:20850. doi: 10.1038/srep20850 (PMC4749989; doi:10.1038/srep20850)
Supplement: Supplementary Information [file srep20850-s1.pdf]

tRNA-Derived Small Non-Coding RNAs in Response to Ischemia Inhibit Angiogenesis

Qing Li[1#], Bin Hu[1#], Guo-wen Hu[2,4], Chun-yuan Chen[1,4], Xin Niu[1], Juan Liu[2], Shu-min Zhou[1], Chang-qing Zhang[1,3*], Yang Wang[1*], Zhi-Feng Deng[2*]

Supplementary Data

Supplementary Table 1. Alignments and statistics of the top 10 tRNA-derived fragments.

Name	Length	Assembly sequence	L_Read (%)	R_Read (%)	Fold Change (R/L)
tRNA1600-ValCAC	33	GTTTCCGTAGTGTAGTGG TTATCACGTTCGCCT	60437.4 (0.521)	1027886 (14.625)	28
tRNA1668-GlyGCC	32	GCATTGGTGGTTCAGTGG TAGAATTCTCGCCT	62573.4 (0.667)	408784.8 (5.704)	8
tRNA7-Gly GCC	32	GCATGGGTGGTTCAGTGG TAGAATTCTCGCCT	5782 (0.074)	81345.8 (1.126)	15
tRNA1610-ValCAC	33	GTTTCCGTAGTGTAGTGG TTATCACGCTCGCCT	545.4 (0.006)	15655 (0.237)	41
tRNA3946-HisGTG	30	GCCGTGATCGTATAGTGG TTAGTACTCTGC	1924.8 (0.018)	13705.2 (0.181)	10
tRNA1631-ValAAC	33	GTTTCCGTAGTGTAGTGG TTATCACATTCGCCT	715.8 (0.006)	12515.4 (0.179)	29.
tRNA22-Gl uCTC	33	TCCCTGGTGGTCTAGTGG TTAGGATTCGGCGCT	273.2 (0.002)	2738.8 (0.038)	16
tRNA7274-GlyCCC	32	GCATTGGTGGTTCAATGG TAGAATTCTCGCCT	167.4 (0.002)	1970.4 (0.032)	18
tRNA3881-GluTTC	33	TCCCACATGGTCTAGCGG TTAGGATTCCTGGTT	122.2 (0.0007)	1691.4 (0.024)	35
tRNA1683-LysTTT	33	GCCCGGATAGCTCAGTCG GTAGAGCATCAGACT	33.6 (0.0003)	1618.4 (0.023)	81

Supplementary Table 2. Sequence analysis of the top 10 tRNA-derived fragments. The most enriched length for tRNA was highlighted.

Sequence (5'-3')	Length	Average reads	
tRNA1600-ValCAC	(nt)	Control	Ischemia
GTTTCCGTAGTGTAGTGGTTATCACGT	27	10.6	79.2
GTTTCCGTAGTGTAGTGGTTATCACGTT	28	24	202.8
GTTTCCGTAGTGTAGTGGTTATCACGTTC	29	16.6	309.8
GTTTCCGTAGTGTAGTGGTTATCACGTTCG	30	24.6	513.2
GTTTCCGTAGTGTAGTGGTTATCACGTTCGC	31	94	578.8
GTTTCCGTAGTGTAGTGGTTATCACGTTCGCC	32	2302.6	20535.6
GTTTCCGTAGTGTAGTGGTTATCACGTTCGCCT	33	60437.4	1027886.4
GTTTCCGTAGTGTAGTGGTTATCACGTTCGCCTC	34	1157.8	5852.4

Sequence (5'-3')	Length	Average reads	
tRNA1668-GlyGCC	(nt)	Control	Ischemia
GCATTGGTGGTTCAGTGGTAGAATTC	26	1.2	43.2
GCATTGGTGGTTCAGTGGTAGAATTCT	27	1	36.8
GCATTGGTGGTTCAGTGGTAGAATTCTC	28	16.2	368.2
GCATTGGTGGTTCAGTGGTAGAATTCTCG	29	29	633
GCATTGGTGGTTCAGTGGTAGAATTCTCGC	30	4121.8	87934.6
GCATTGGTGGTTCAGTGGTAGAATTCTCGCC	31	6970.2	68232.4
GCATTGGTGGTTCAGTGGTAGAATTCTCGCCT	32	62573.4	408784.8
GCATTGGTGGTTCAGTGGTAGAATTCTCGCCTG	33	538.4	11164.8
GCATTGGTGGTTCAGTGGTAGAATTCTCGCCTGC	34	616.4	2582.8

Sequence (5'-3')	Length	Average reads	
tRNA7-GlyGCC	(nt)	Control	Ischemia
GCATGGGTGGTTCAGTGGTAGAATTC	26	3.6	29.6
GCATGGGTGGTTCAGTGGTAGAATTCT	27	0.2	22.2
GCATGGGTGGTTCAGTGGTAGAATTCTC	28	5.4	168.8
GCATGGGTGGTTCAGTGGTAGAATTCTCG	29	1.6	103.8
GCATGGGTGGTTCAGTGGTAGAATTCTCGC	30	51.4	3597.8
GCATGGGTGGTTCAGTGGTAGAATTCTCGCC	31	234.6	6547.2
GCATGGGTGGTTCAGTGGTAGAATTCTCGCCT	32	5782	81345.8
GCATGGGTGGTTCAGTGGTAGAATTCTCGCCTG	33	90.8	4565.8
GCATGGGTGGTTCAGTGGTAGAATTCTCGCCTGC	34	19.2	259

Sequence (5'-3')	Length	Average reads	
tRNA1610-ValCAC	(nt)	Control	Ischemia
GTTTCCGTAGTGTAGTGGTTATCACG	26	9.6	41.8
GTTTCCGTAGTGTAGTGGTTATCACGC	27	0.2	4.2
GTTTCCGTAGTGTAGTGGTTATCACGCT	28	0	4.4

GTTTCCGTAGTGTAGTGGTTATCACGCTC	29	15.4	39
GTTTCCGTAGTGTAGTGGTTATCACGCTCG	30	1.8	17
GTTTCCGTAGTGTAGTGGTTATCACGCTCGC	31	0.4	14.8
GTTTCCGTAGTGTAGTGGTTATCACGCTCGCC	32	50.2	609.2
GTTTCCGTAGTGTAGTGGTTATCACGCTCGCCT	33	545.4	15655
GTTTCCGTAGTGTAGTGGTTATCACGCTCGCCTC	34	9.6	181.4

Sequence (5'-3')	Length	Average reads	
tRNA3946-HisGTG	(nt)	Control	Ischemia
GCCGTGATCGTATAGTGGTTAGTACT	26	35.8	138.8
GCCGTGATCGTATAGTGGTTAGTACTC	27	44.2	152.6
GCCGTGATCGTATAGTGGTTAGTACTCT	28	108.2	596
GCCGTGATCGTATAGTGGTTAGTACTCTG	29	234	1576.8
GCCGTGATCGTATAGTGGTTAGTACTCTGC	30	1924.8	13705.2
GCCGTGATCGTATAGTGGTTAGTACTCTGCG	31	416.6	4486
GCCGTGATCGTATAGTGGTTAGTACTCTGCGT	32	20.4	207.8
GCCGTGATCGTATAGTGGTTAGTACTCTGCGTT	33	292	1425.8
GCCGTGATCGTATAGTGGTTAGTACTCTGCGTTG	34	26.6	279.8

Sequence (5'-3')	Length	Average reads	
tRNA1631-ValAAC	(nt)	Control	Ischemia
GTTTCCGTAGTGTAGTGGTTATCACATTC	29	2.2	12.8
GTTTCCGTAGTGTAGTGGTTATCACATTCGC	31	0.2	7
GTTTCCGTAGTGTAGTGGTTATCACATTCGCC	32	21.6	236.6
GTTTCCGTAGTGTAGTGGTTATCACATTCGCCT	33	715.8	12515.4

Sequence (5'-3')	Length	Average reads	
tRNA22-GluCTC	(nt)	Control	Ischemia
TCCCTGGTGGTCTAGTGGTTAGGATT	26	0.4	39.4
TCCCTGGTGGTCTAGTGGTTAGGATTC	27	481	21.6
TCCCTGGTGGTCTAGTGGTTAGGATTCG	28	16.2	188.2
TCCCTGGTGGTCTAGTGGTTAGGATTCGG	29	8.2	45.8
TCCCTGGTGGTCTAGTGGTTAGGATTCGGC	30	52.8	581.4
TCCCTGGTGGTCTAGTGGTTAGGATTCGGCG	31	45	266.8
TCCCTGGTGGTCTAGTGGTTAGGATTCGGCGC	32	14.8	416
TCCCTGGTGGTCTAGTGGTTAGGATTCGGCGCT	33	273.2	2738.8
TCCCTGGTGGTCTAGTGGTTAGGATTCGGCGCTC	34	284	649.6

Sequence (5'-3')	Length	Average reads	
tRNA7274-GlyCCC	(nt)	Control	Ischemia
GCATTGGTGGTTCAATGGTAGAATTCTCGC	30	6.8	56.2
GCATTGGTGGTTCAATGGTAGAATTCTCGCC	31	38.6	413.8
GCATTGGTGGTTCAATGGTAGAATTCTCGCCT	32	167.4	1970.4

GCATTGGTGGTTCAATGGTAGAATTCTCGCCTC	33	7	66.8
GCATTGGTGGTTCAATGGTAGAATTCTCGCCTCC	34	5	38.6

Sequence (5'-3')	Length	Average reads	
tRNA3881-GluTTC	(nt)	Control	Ischemia
TCCCACATGGTCTAGCGGTTAGGATT	26	1.4	19
TCCCACATGGTCTAGCGGTTAGGATTC	27	2	5.8
TCCCACATGGTCTAGCGGTTAGGATTCC	28	3.4	148.4
TCCCACATGGTCTAGCGGTTAGGATTCCT	29	220.4	882.6
TCCCACATGGTCTAGCGGTTAGGATTCCTG	30	10	740.8
TCCCACATGGTCTAGCGGTTAGGATTCCTGG	31	21.6	351.2
TCCCACATGGTCTAGCGGTTAGGATTCCTGGT	32	35.2	236
TCCCACATGGTCTAGCGGTTAGGATTCCTGGTT	33	122.2	1691.4
TCCCACATGGTCTAGCGGTTAGGATTCCTGGTTT	34	1.4	115.8

Sequence (5'-3')	Length	Average reads	
tRNA1683-LysTTT	(nt)	Control	Ischemia
GCCCGGATAGCTCAGTCGGTAGAGCATC	28	7.6	46.8
GCCCGGATAGCTCAGTCGGTAGAGCATCA	29	3.4	21
GCCCGGATAGCTCAGTCGGTAGAGCATCAG	30	9	21.4
GCCCGGATAGCTCAGTCGGTAGAGCATCAGA	31	27.8	49.2
GCCCGGATAGCTCAGTCGGTAGAGCATCAGAC	32	33.4	939.8
GCCCGGATAGCTCAGTCGGTAGAGCATCAGACT	33	33.6	1618.4
GCCCGGATAGCTCAGTCGGTAGAGCATCAGACTT	34	2.6	22.4

Supplementary figure 1

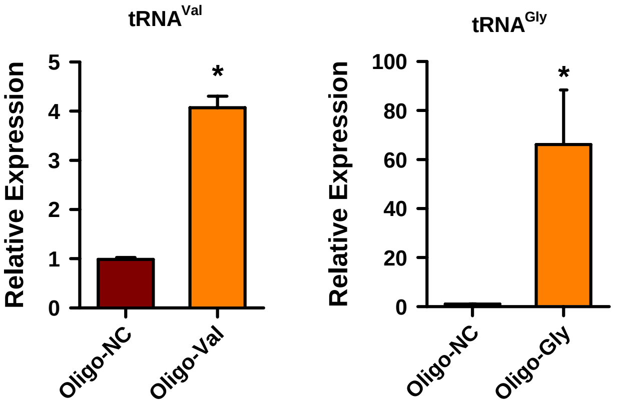

Supplementary figure 1. HUVEC were transfected with Oligo-NC, Oligo-Val, and Oligo-Gly for 24hrs and the expression level of $tRNA^{Val}$- and $tRNA^{Gly}$-derived small RNAs was determined by qPCR analysis. *$P < 0.05$

Supplementary figure 2

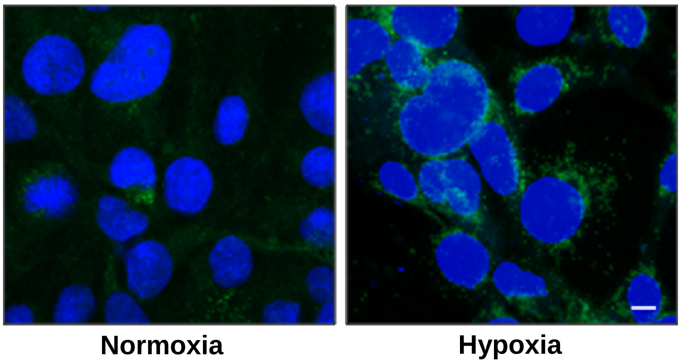

Supplementary figure 2. ECs were subjected to normoxia or hypoxia (1% oxygen) for 48 hrs, and immunostaining showed that the expression of ANG (green) was up-regulated upon hypoxia stimulation, and were most located in cytoplasm. Scale bar: 10μm.
